# Green synthesis of silver nanoparticles using Saudi *Xanthium strumarium* extract: anticancer potential against breast and lung cancer cells

**DOI:** 10.3389/fphar.2025.1653711

**Published:** 2025-11-07

**Authors:** Hanan Aati, Jawaher Al-Qahtani, Areej Al-Taweel, Nida N. Farshori, Raha Orfali, Shagufta Perveen

**Affiliations:** 1 Department of Pharmacognosy, College of Pharmacy, King Saud University, Riyadh, Saudi Arabia; 2 Department of Bacteriology, University of Wisconsin-Madison, Madison, WI, United States

**Keywords:** *Xanthium strumarium*, green synthesis, silver nanoparticles, cytotoxicity, morphological changes, ROS generation

## Abstract

**Introduction:**

This study reports *Xanthium strumarium *aqueous extract-mediated synthesis of silver nanoparticles and mechanistic evaluation of their antiproliferative effects against breast (MCF-7) and lung (A-549) cancer cells.

**Methods:**

The synthesized XT-AgNPs were analyzed in terms of their size, structure, shape, and morphology using various analytical techniques. UV/Vis spectroscopy revealed a distinct peak at 447 nm, indicating the successful synthesis of AgNPs. SEM/EDX and TEM analyses confirmed that the XT-AgNPs had spherical shape and exhibited a uniform size distribution. XRD analysis verified that XT-AgNPs exhibited a face-centered cubic (FCC) crystalline structure, with a calculated crystalline size of 27.90 nm. Additionally, the cytotoxic potential of XT-AgNPs were examined on MCF-7 and A-549 cancer cells using MTT and NRU assays. Cell death was assessed through morphological analysis, while the reactive oxygen species generation was evaluated using DCFH-DA dye.

**Results and Discussion:**

XT-AgNPs demonstrated notable cytotoxic effects on MCF-7 and A-549 cells with IC_50_ values of 44.3 and 57.4 μg/mL, respectively. Morphological and DCFH-DA analyses revealed the effect of XT-AgNPs in reducing the cell proliferation and inducing ROS generation. GC/MS analysis was conducted to identify the phytochemicals present in the extract which showed 29 peaks of phytoconstituents. These 27 phytoconstituents were further analyzed by *in silico* studies, molecular docking study was carried out for all phytoconstituents, and compounds having maximum binding affinity were analyzed for their ADMET profile. Overall, the results highlight the anticancer properties of XT-AgNPs, indicating their potential as an effective agent in human breast and lung cancer management.

## Introduction

Cancer is an enduring global health challenge characterized by an uncontrolled growth of cells. Its development is often linked to factors such as lifestyle changes, nutrition, and exposure to air pollution and other carcinogens (Das et al., 2023). As cancer cells undergo uncontrolled growth and proliferation, they become genetically unstable (Williams and Stoeber, 2012). The elevated death rate is attributed to a poor prognosis for many types of cancer, posing a significant concern for public health. Conventional cancer treatments are effective in eliminating tumor cells, but they often come with various aftereffects (Anand et al., 2023). Hence, it is essential to explore alternative strategies for cancer treatment. Recently, there has been significant interest in incorporating nanotechnology into cancer treatment (Dessale et al., 2022). Nanotechnology is a wide-ranging field of research with numerous applications aimed at improving quality of life (Malik et al., 2023). Over the past few decades, it has significantly influenced the diagnosis and treatment of cancer, owing to its unique capabilities in diagnosis, imaging, drug delivery, and enhancing treatment effectiveness (Rashidi et al., 2024). Nanomedicines have emerged as a prominent research field, with scientists focused on developing safe, effective, and more affordable drugs that are also less toxic to address diseases such as cancer (Chehelgerdi et al., 2023). AgNPs are particularly noteworthy as their extensive use in biotechnology and biomedical fields (Meher et al., 2024). AgNPs find significant applications in medical and cosmetic products, as well as in the treatment of water, and are also used in pesticides and household items (Bhardwaj et al., 2021). Following significant interest in AgNPs, researchers are exploring a range of eco-friendly methods for nanoparticle synthesis (Fahim et al., 2024). Silver nanoparticles (AgNPs) have traditionally been synthesized using chemical and physical methods. However, these chemical methods often involve toxic substances that can be harmful to living organisms. To address this issue, AgNPs are now being synthesized using biological methods grounded in green chemistry, which help minimize the use of harmful chemicals (Ying et al., 2022). The green synthesis of AgNPs from various natural resources is an emerging area within green nanotechnology (Akhter et al., 2024). The green chemistry of synthetic approaches employing biological methods, such as enzymes, microorganisms, and plant extracts, plays a vital role in synthesis of AgNPs (Sharma et al., 2022). Among biological methods, synthesizing AgNPs via plant extracts is considered the most ecofriendly alternative to traditional chemical and physical methods (Yarrappagaari et al., 2020). Additionally, this approach can be easily scaled up for large-scale production of AgNPs. Recent studies showed that plants such as *Hibiscus sabdariffa* (Ahirwar et al., 2024), *Rubus fruticosus* (Khan et al., 2024), *Rhus chinensis Mill* (Bhusal et al., 2024)*, Citrus* aurantiifolia (Mustapha et al., 2023), *Ocimum kilimandscharicum* (Ouandaogo et al., 2024); *Avicennia marina (Mangrove)* (Gramah et al., 2024), and *Aloe fleurentiniorum* (Jamil et al., 2024) have been effectively screened for their potential in synthesizing silver nanoparticles from plant extracts. Numerous researchers have reported strong anticancer activities associated with green synthesized silver nanoparticles (Jabeen et al., 2021; Bhat et al., 2024; Gaffar et al., 2024; Tanwar et al., 2024). The green synthesized AgNPs have also been reported to possess cytotoxic potential against various cancer cell lines such as, *Moringa peregrina* against human breast and colon cancer cells (Al Baloushi et al., 2024), *Ocimum basilicum* L. against cervical HeLa cancer cells (Qamar et al., 2024), *Galega officinali against* prostate DU-145, colon HT-29 (Entezari Heravi et al., 2018), and *Phoenix dactylifera* against lung A-549 (Farshori et al., 2022). The eco-friendly synthesis methods and anticancer potentiality of silver nanoparticles have inspired the current study. This research article concentrates on the green synthesis of AgNPs using *Xanthium strumarium* extract and its anticancer potential on human breast (MCF-7) and lung (A-549) cancer cells. *Xanthium strumarium* L. (commonly referred to as the rough cocklebur) is a kind of annual plant, affiliated to Asteraceae family. The plant is believed to have certain medicinal properties and has been utilized in traditional medicine in South Asia as well as in traditional Chinese medicine (Kamboj and Saluja, 2010). Several biological activities of *X. strumarium* have been revealed such as antiulcerogenic, anthelmintic actions (Sharma, 2003), anti-inflammatory, diuretic, leishmanicidal, antifungal properties (Kamboj and Saluja, 2010), and a remarkable sedative effect on brain too (Mandal et al., 2001). The presence of these phytochemicals suggests that the plant has multifaceted mechanisms of action, which can be harnessed for medicinal purposes. In light of the enormous potential of the plants, we report for the first time, the green synthesis of AgNPs using *Xanthium strumarium* grown in Saudi Arabia. Furthermore, this study investigates the anticancer activity of the green synthesized AgNPs (XT-AgNPs) derived from *X. strumarium* extract against the human breast (MCF-7) and lung (A-549) carcinoma cell lines.

## Materials and methods

### Plant materials

Fresh *Xanthium* plants were gathered from Najran, located in the southern part of the Kingdom of Saudi Arabia between October and November 2020. Fresh leaves were collected at the vegetative stage. Only healthy, mature leaves were selected. The guidelines and regulations were strictly adhered to during the sample collection. Dr. Rajakrishnan Rajagopal from the College of Sciences, KSU, taxonomically identified the collected plant materials. The voucher specimens were preserved at the Herbarium Centre under the accession number 22677.

### Preparation of plant extract

Leaves were shade-dried at room temperature (25 °C ± 2 °C) for 7 days until constant weight was obtained. The dried material was ground into a fine powder, and 10 g of dry mass was used for extraction. The powder was mixed with 100 mL of distilled water (solid-to-liquid ratio 1:10 w/v) and subjected to continuous stirring in the dark to prevent photodegradation. The pH was measured as 6.2. The extract was first filtered through Whatman No. 1 paper, followed by passage through a 0.22 μm membrane filter to remove particulates and microbial contaminants. For silver nanoparticle synthesis, the extract was used immediately after preparation. When storage was necessary, aliquots were kept in sterile amber glass vials at 4 °C for no longer than 72 h.

### GC-MS

An Agilent 5977A GC System with an HP-5MS GC column, 30 m × 0.25 mm × 0.25 μm (with a maximum temperature of 350 °C) was used to profile the extract sample using GC-MS. Before the analysis, the aqueous extract (2 mL) was lyophilized and reconstituted in 1 mL of methanol prior to GC–MS injection. This system was integrated with an Agilent 5977A Series MSD system. Ultra-high-purity helium (99.99%) served as the carrier gas, maintaining a constant flow rate of 1.2 mL/min. The injection, transfer line, and ion source temperatures were set at 310 °C, with an ionization energy of 70 eV. The oven was automated to ramp at a rate of 5 °C/min from 60 °C (which was held for 7 min) to 310 °C. A 50:1 split ratio was used, with a 1 μL injection volume. During the data collection process, full-scan mass spectra between 35 and 650 amu were captured. Chemical compounds were tentatively identified and classified based on the most closely matching fragmentation patterns and GC retention times. Using MassHunter GC/MS Acquisition, mass spectra were compared to standards found in NIST-02, RTLPEST3.L and SWGDRUG 3.9.L mass spectrum libraries (Aati et al., 2022).

### Green synthesis of AgNPs

AgNP synthesis was conducted using a one-step method (Farshori et al., 2022). A 1 mM AgNO_3_ stock solution was prepared using analytical-grade silver nitrate. For each synthesis, 10 mL of plant extract was added to 90 mL of AgNO_3_ solution, yielding a final reaction volume of 100 mL and a final Ag^+^ concentration of 0.9 mM. A visible color change from pale yellow to dark brown occurred within 15 min of heating at 80 °C and the UV–Vis spectra confirmed the appearance of the characteristic SPR band after ∼30 min. Three indispensable controls were performed: AgNO_3_ solution without extract, Plant extract without AgNO_3_ and AgNO_3_ + extract at room temperature (25 °C ± 2 °C). To separate the nanoparticles, the mixture was centrifuged at 10,000 rpm for 10 min. The resulting AgNP pellet was collected and cleaned with deionized H_2_O to eliminate residual phytochemicals and silver ions. Finally, the green-synthesized silver nanoparticles (XT-AgNPs) were lyophilized and stored in a cool, dry place for future applications.

### Characterization of AgNPs

#### Evaluation of chemical characteristics using FT-IR technique

XT-AgNPs were synthesized using *Xanthium strumarium* extract, potentially leading to the presence of associated biomolecules. FT-IR analysis (Perkin Elmer) was performed to identify these biomolecules by detecting moieties. For this, the sample was prepared using the KBr pellet and spectrum was recorded within the range of 4,000–650 cm^−1^.

#### UV-vis examination

UV-vis analysis was conducted using a spectrophotometer (Shimadzu, Japan) to monitor the formation of XT-AgNPs in the suspension, with a wavelength range spanning from 190 to 800 nm.

#### Evaluation of morphology

The morphology and size of XT-AgNPs were examined using SEM (scanning electron microscope (JEOL, JSM-7600F) equipped with EDX) and TEM (transmission electron microscope (JEOL, JEM-2100F) operating at 200 keV). For TEM, a drop of nanoparticle dispersion (0.1 mg/mL) was placed onto a carbon-coated copper grid and allowed to dry at room temperature; no additional staining was applied. TEM micrographs were recorded at appropriate magnifications with visible scale bars. For SEM, nanoparticle samples were drop-cast onto a silicon wafer, air-dried, and coated with a thin gold layer (∼5 nm) to enhance conductivity. Particle size measurements were performed on >200 nanoparticles using ImageJ software, and results are reported as mean ± standard deviation with size range. Histograms of particle size distribution are provided.

#### DLS analysis

The size distribution in aqueous suspension was determined using dynamic light scattering (DLS) with a Zetasizer instrument (Malvern, UK). Briefly, XT-AgNPs were suspended in water, sonicated, and analyzed at 25 °C.

#### X-ray diffraction analysis

X-ray diffraction (XRD) with Cu radiation was employed to analyze the crystalline structure of XT-AgNPs under the conditions of 30 kV; 40 mA; Kα radiation (1.54430 Å). For structural characterization, the reaction mixture was centrifuged at 12,000 rpm for 20 min. The pellet was washed three times with distilled water to remove unbound biomolecules, then lyophilized at −50 °C under vacuum for 24 h to obtain a fine, dry powder for XRD analysis.

### *In vitro* anticancer assays

#### Cell culture and treatment

MCF-7 and A-549 cancer cells were acquired from ATCC and maintained in DMEM with 10% FBS, and antibiotic solution (100 μg/mL of streptomycin and 100 units/mL of penicillin; Gibco Life Technologies, USA) in a CO_2_ incubator (5% CO_2_, 95% relative humidity) at 37 °C (Thermo Scientific, USA). Before conducting experiments, cell viability was assessed by trypan blue test, ensuring >95% viability for the study. A solution of synthesized AgNPs (10 mg/mL) was made in PBS, then serially diluted in fresh complete medium to achieve the desired concentrations for cell treatment.

#### MTT assay

The cytotoxicity of XT-AgNPs was evaluated using the MTT assay, as previously described (Siddiqui et al., 2008). In brief, cells were exposed to different concentrations of XT-AgNPs, XT extract, and commercial AgNPs for 24 h. After incubation, 10 µL of MTT (5 mg/mL) was added to each well, and subsequently, plates were incubated at 37 °C for an additional 4 h. The formazan product formed was dissolved in 200 µL of dimethyl sulfoxide (DMSO) with gentle shaking. The absorbance at 550 nm was recorded with a microplate reader (Multiskan EX, Thermo Scientific, Finland).

#### NRU assay

Cytotoxicity of synthesized XT-AgNPs was also assessed using the neutral red uptake (NRU) assay, following previously established protocols (Siddiqui et al., 2008). Both MCF-7 and A-549 cells were exposed to varying concentrations of XT-AgNPs and incubated for 24 h. After exposure, the cells were incubated with neutral red medium (50 μg/mL) for additional 3 h. Subsequently, the cells were washed, and the dye was extracted using a solution of 50% ethanol, 1% acetic acid, and 49% double-distilled water. The absorbance was then recorded at 550 nm. Control groups (without XT-AgNPs) were also run parallel under identical conditions. All the experiments were performed in triplicate. Afterward, percentage cell viability in treated groups was determined according to following equation considering control group as 100%.

$$\%\text{ cell viability}=\frac{\text{Mean}\;\left(\text{OD}\;\text{treated}\;–\;\text{OD}\;\text{blank}\right)\times 100}{\text{Mean}\;\left(\text{OD}\;\text{control}\;–\;\text{OD}\;\text{blank}\right)}$$

(Where OD treated denotes the optical density (OD) in treated group, OD control denotes the OD in DMEM cell culture medium, and OD blank denotes the OD measured in the solution without any cells).

#### Morphological changes

The shape and morphology of MCF-7 and A-549 cells was assessed using a phase contrast microscopy. Both cells were exposed to variable concentrations of XT-AgNPs for 24 h. Then, the treated and control cells were examined under microscope (CKX41, Olympus, Japan) and photographed using the attached camera for live imaging.

#### ROS generation

DCF-DA was employed to assess the intracellular production of reactive oxygen species (ROS) (Farshori et al., 2022). Upon passive entry into the cells, DCFH-DA reacts with ROS to produce the highly fluorescent compound DCF. MCF-7 and A-549 cancer cells were seeded inside 24-well plates and kept to adhere over 24 h. After treatment, the cells were incubated for 60 min at 37 °C with a 20 µM DCFH-DA working solution. Following incubation, cell images were grabbed under a fluorescence microscope.

### *In silico* studies

#### Molecular docking

When researching drug design with computer assistance, it is an extremely beneficial tool. To begin, protein resolutions for P53 (2OCJ), BCL2 (2W3L), EGFR (3POZ), and HER2 (1N8Z) were gained from Protein Data Bank. Discovery Studio 2021 was used to prepare proteins. Water molecules, heteroatoms and chains from protein molecules were removed, with the exception of the A chain. Following that, polar hydrogen molecules were supplemented to protein, and the resultant file was saved as a Protein Data Bank file. The secondary metabolites were selected using the GC-MS analytical method and obtained from PubChem database in SDF (structured data file). Following that, the macromolecule option was completed, and the protein molecule was uploaded to the PyRx program. Following that, ligands were uploaded into PyRx using the openbabel option. After that, the ligand was minimized and transferred to PDBQT format. To analyze the binding results, a precisely measured grid box was created, and the ligand-protein interaction was initiated. Ultimately, the interactions were analysed using Discovery Studio (Tabassum et al., 2022). To ensure reproducibility and reliability, docking was performed with defined quality criteria, including specification of the chosen active/binding site for each protein, the use of positive and negative controls, assessment of pose reproducibility (RMSD <2 Å), and interpretation of docking results within established score thresholds. These criteria ensured consistency and minimized false-positive interpretations.

#### ADME study

ADME properties of top docked bioactive compounds were evaluated through online SwissADME tool available at http://www.swissadme.ch/.

#### Toxicity evaluation

For the top-docked compounds, the toxicity was assessed via online program PROTOX II, available at https://tox-new.charite.de/.

### Statistical analysis

The experimental data are presented as the mean ± S.D., with n = 3 per group. The data were analyzed one-way by ANOVA, followed by Donnette’s *post hoc* multiple comparison test using Origin6.1 software, where a p value of <0.05 was regarded as statistically significant. All the experiments were performed in triplicate.

## Results and Discussion

### Tentative identification of phytoconstituent using GC-MS analysis

**FIGURE 1:**
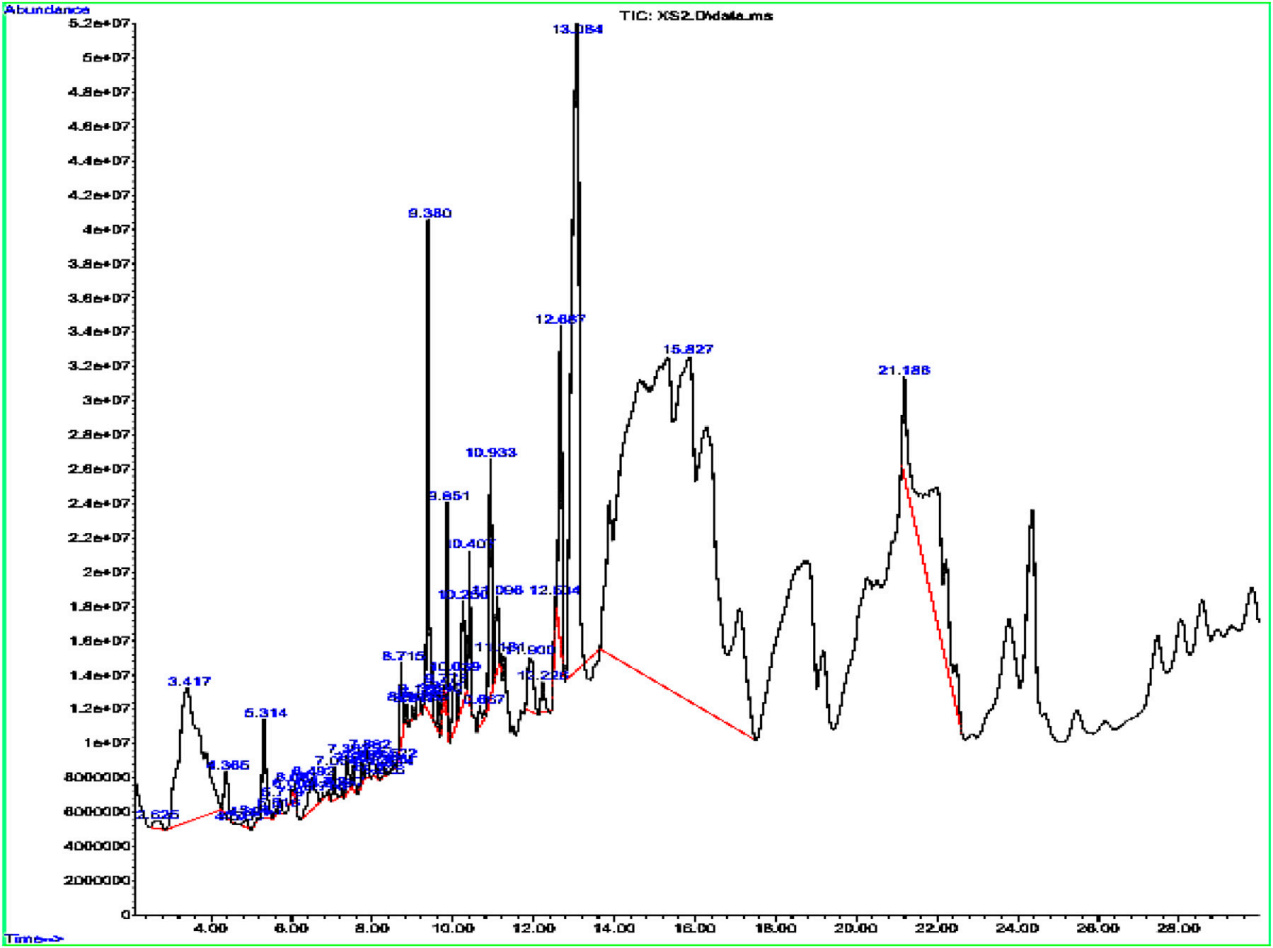
Peaks of the tentatively identified compounds by GC-MS.

The GC–MS profiling of the plant extract revealed a chemically diverse mixture of volatile and semi-volatile constituents spanning phenethylamines, phenolic derivatives, fatty acids, esters, terpenoids, alkaloids, and nucleosides (Figure 1; Table 1). Among the early eluting compounds, 3-hydroxy-N-methylphenethylamine (RT 2.62 min) represents a phenethylamine derivative, while 2,6-dimethoxyphenol and phenol are simple aromatic compounds that may contribute antioxidant activity of plant extract (Schirmann et al., 2018). The detection of 4-(2,6,6-trimethylcyclohexa-1,3-dienyl) but-3-en-2-one suggests the presence of phenylpropanoid derivatives. Medium-retention compounds included fatty acids and their esters such as hexanoic acid, stearic acid, palmitic acid, tetradecanoic acid, and several methyl esters (hexadecanoic acid methyl ester, pentadecanoic acid methyl ester, and linoleic/linolenic acid methyl esters). Isopropyl palmitate, detected as a fatty acid ester while several terpenoid structures were tentatively identified, including longifolene, neophytadiene, phytol, and partially hydrogenated naphthalene derivatives. Phytol, a diterpene alcohol (Basit et al., 2023) and sesquiterpenes such as neophytadiene and longifolene are were also tentatively identified in the extract sample. Other notable constituents included p-tyramine (a biogenic amine with neuromodulatory effects), oxirane derivatives, and cyclopentenone structures, which may act as electrophilic bioactive molecules. Guanosine, a nucleoside detected at longer retention times, was identified tentatively; however, due to its high polarity and low volatility, the reliability of its detection in non-derivatized extracts need the further confirmation through running standard. This kind of polarity variation among the tentatively identified compounds have been seen previously in the GC-MS analysis of different plant extract all with non-derivatized extract sample preparation (Basit et al., 2022a; Basit et al., 2022b; Ahmad et al., 2022). Overall, the tentative metabolite profile indicates that the extract is enriched in terpenoids and fatty acid derivatives, with additional contributions from phenolics, alkaloids, and amines. While the GC–MS results provide a preliminary indication of phytochemical diversity, future work employing derivatization and complementary LC–MS/MS analysis will be required to confirm polar metabolites such as sugars and nucleosides.

**TABLE 1:** Details of the tentatively identified compounds in GC-MS analysis of the extract sample.

SR #	Component RT	Area %	Compound name	Formula	Molecular weight	Chemical class
1	2.625	1.16	3-Hydroxy-Nmethylphenethylamine	C_9_H_13_O	151.21	Phenethylamine
2	4.365	1.48	2,6-dimethoxy-phenol	C_8_H_10_O_3_	154.16	Methoxyphenols
3	5.314	1.00	4-(2,6,6-Trimethylcyclohexa-1,3-dienyl) but-3-en-2-one	C_13_H_18_O	190.28	Phenylpropanoids
4	6.492	1.59	Phenol	C_6_H_6_O	94.11	Aromatic compounds
5	7.054	1.22	Hexanoic acid	C_6_H_12_O_2_	116.16	Carboxylic acids
6	7.362	1.29	Beta-D-Glucopyranose,4-O-Beta-D-galactopyranosyl	C_12_H_22_O_11_	342.3	Glycosides
7	7.537	1.14	Stearic Acid	C_18_H_36_O_2_	284.5	Fatty acid
8	7.737	1.14	4-Isopropyl-1,6-dimethyl-1,2,3,4-tetrahydronaphthalene	C_15_H_22_	202.33	Terpenoids
9	7.882	1.16	Pilocarpine	C_11_H_16_N_2_O_2_	208.26	Alkaloid
10	8.715	1.53	Longifolene-(V4)	C_15_H_24_	204.35	Sesquiterpenes
11	9.126	1.17	1-Octadecene	C_18_H_36_	252.5	Alkenes
12	9.380	2.85	Neophytadiene	C_20_H_38_	278.5	Sesquiterpenoids
13	9.640	1.13	Tetradecanoic acid	C_14_H_28_0_2_	228.37	Fatty acid
14	9.851	1.83	Oxirane, hexadecyl-	C_18_H_36_O	268.5	Epoxide
15	10.039	1.50	2,4(1H,3H)-Pyrimidinedione, 5-methyl-	C_5_H_6_N_2_O_2_	126.11	Pyrimidinedione
16	10.250	1.87	2-Cyclopenten-1-one, 3-methyl-2-(2,4-pentadienyl)-, (Z)-	C_11_H_14_O	162.23	Cyclopentenone
17	10.407	1.66	Hexadecanoic acid, methyl ester	C_17_H_34_O_2_	270.5	Fatty acid ester
18	10.667	1.18	Pentadecanoic acid, 14-methyl ester	C_17_H_34_O_2_	270.5	Fatty acid esters
19	10.933	1.76	palmitic acid	C_16_H_32_O_2_	256.42	Saturated fatty acids
20	11.096	1.53	p-Tyramine	C_8_H_11_NO	137.18	Amine
21	11.990	1.84	Isopropyl Palmitate	C_19_H_38_O_2_	298.5	Fatty acid ester
22	12.226	1.26	cis-10-Heptadecenoic acid	C_17_H_32_O_2_	268.4	Fatty acid
23	12.534	1.25	9,12-Octadecadienoic acid (Z,Z)-,methyl ester	C_19_H_34_O_2_	294.5	Fatty acid ester
24	12.667	2.44	9,12,15-Octadecatrienoic acid, methyl ester, (Z,Z,Z)-	C_19_H_32_O_2_	292.5	Fatty acid ester
25	13.084	11.64	Phytol	C_20_H_40_O	296.5	Terpenoids
26	15.827	12.00	Naphthalene, 1,2,3,4,4a,5,6,8a-oct ahydro-4a,8-dimethyl-2-(1-methylet henyl)-, [2R-(2.alpha.,4a.alpha.,8 a.beta.)]-	C_15_H_24_	204.35	Terpene and aromatic hydrocarbon
27	21.186	8.72	Guanosine	C_10_H_13_N_5_O_5_	283.24	Nucleosides

### Green synthesis of AgNPs

**FIGURE 2:**
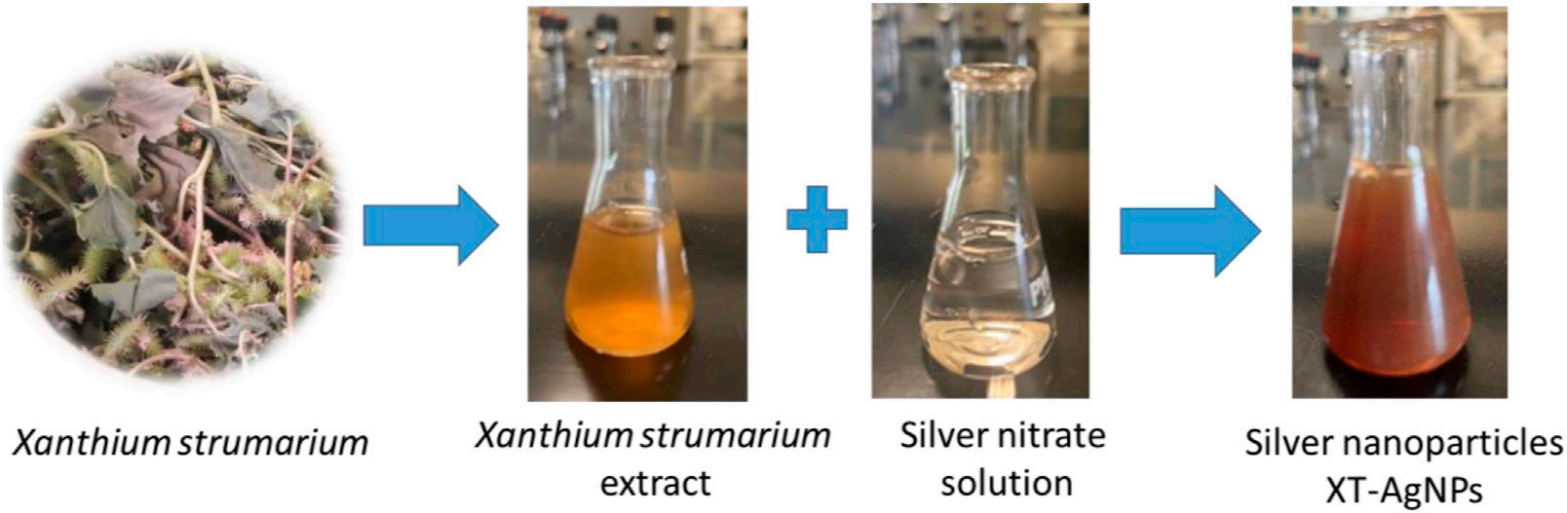
Formation of silver nanoparticles (XT-AgNPs) using *Xanthium strumarium* extract.

The synthesis of nanoparticles begins by adding the plant extract with 1 mM AgNO_3_ solution. The solution gradually transitions from colorless to pale yellow and eventually to dark brown, signifying the formation of AgNPs, as depicted in Figure 2. This color shift is attributed to surface plasmon resonance, a unique optical property of noble metals. The formation of AgNPs was further validated through various analytical techniques.

### UV-visible spectroscopy

**FIGURE 3:**
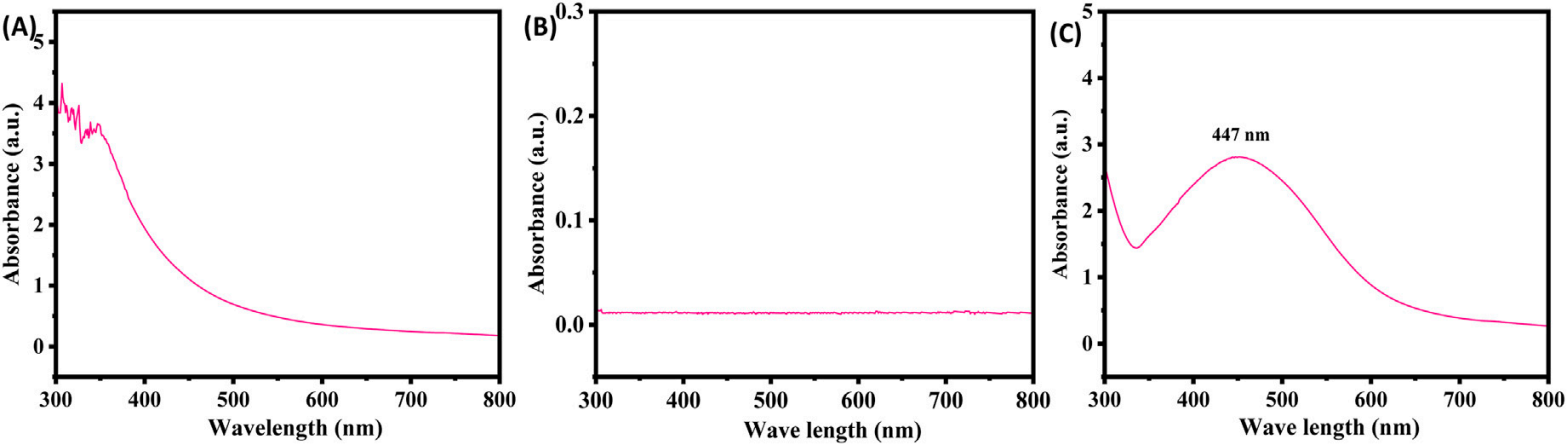
UV-Visible spectra of **(A)** plant extract, **(B)** silver nitrate, and **(C)** green synthesized silver nanoparticles (XT-AgNPs) using *Xanthium strumarium* extract.

UV-Visible spectrophotometric analysis was performed as a preliminary investigation into the synthesis of silver nanoparticles (AgNPs). A drastic color change occurred after mixing plant extract with AgNPs, transitioning from light yellow to dark brown, which confirms production of the nanoparticles. The absorbance of the solution was monitored over the course of a week. Spectral analysis revealed no peak for plant extract (Figure 3A) and silver nitrate (Figure 3B) and a peak for the XT-AgNPs at 447 nm, which was the most pronounced peak (Figure 3C), and it remained stable for several days, with no further rise in absorption. Comparable findings have been reported by Zhang et al. (2022).

### FTIR

**FIGURE 4:**
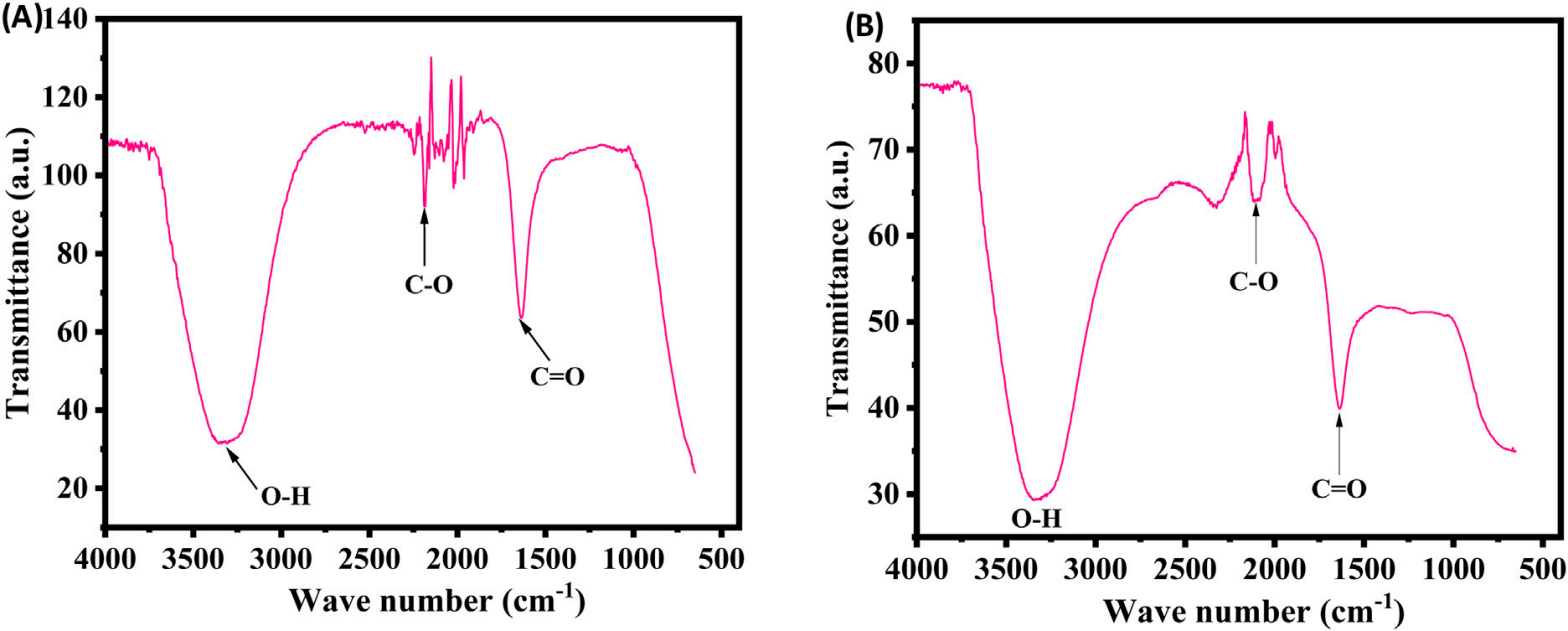
FTIR spectra of **(A)** plant extract and **(B)** Green synthesized XT-AgNPs.

FTIR measurements were performed to determine the key functional groups in the extract and their potential roles in the stabilization and synthesis of XT-AgNPs. The spectra for both the extract and the synthesized XT-AgNPs are displayed in Figure 4. The spectra showed multiple peaks, reflecting the complex nature of the biological material. A distinctive large band in the middle of the spectrum, between 3,200–3,400 cm^−1^, attributes to the O-H group extending vibrations, most likely from an alcohol or phenolic structure (Nandiyanto et al., 2019). In addition, there is a narrow intense absorption band near 1710 cm^−1^, that is a signature of the C=O bond stretching vibrational mode; hence, there is a carbonyl functionality present in the molecule such as, e.g., ketone, aldehyde, ester however not exhaustively (Ezealisiji et al., 2017). The bands within the 2,100–2,250 cm^−1^ range arise from the stretching of carbon-carbon triple bond, characteristic of alkynes or the carbon-nitrogen triple bond, of nitriles. “The observed shifts of the O–H (3,200–3,400 cm^−1^) and C=O (∼1710 cm^−1^) bands, together with attenuations in the 2,100–2,250 cm^−1^ region, indicate adsorption of extract phytochemicals onto the AgNP surface, thereby acting as capping agents that drive nanoparticle formation and stabilization” (Sancheti et al., 2023).

### XRD examination

**FIGURE 5:**
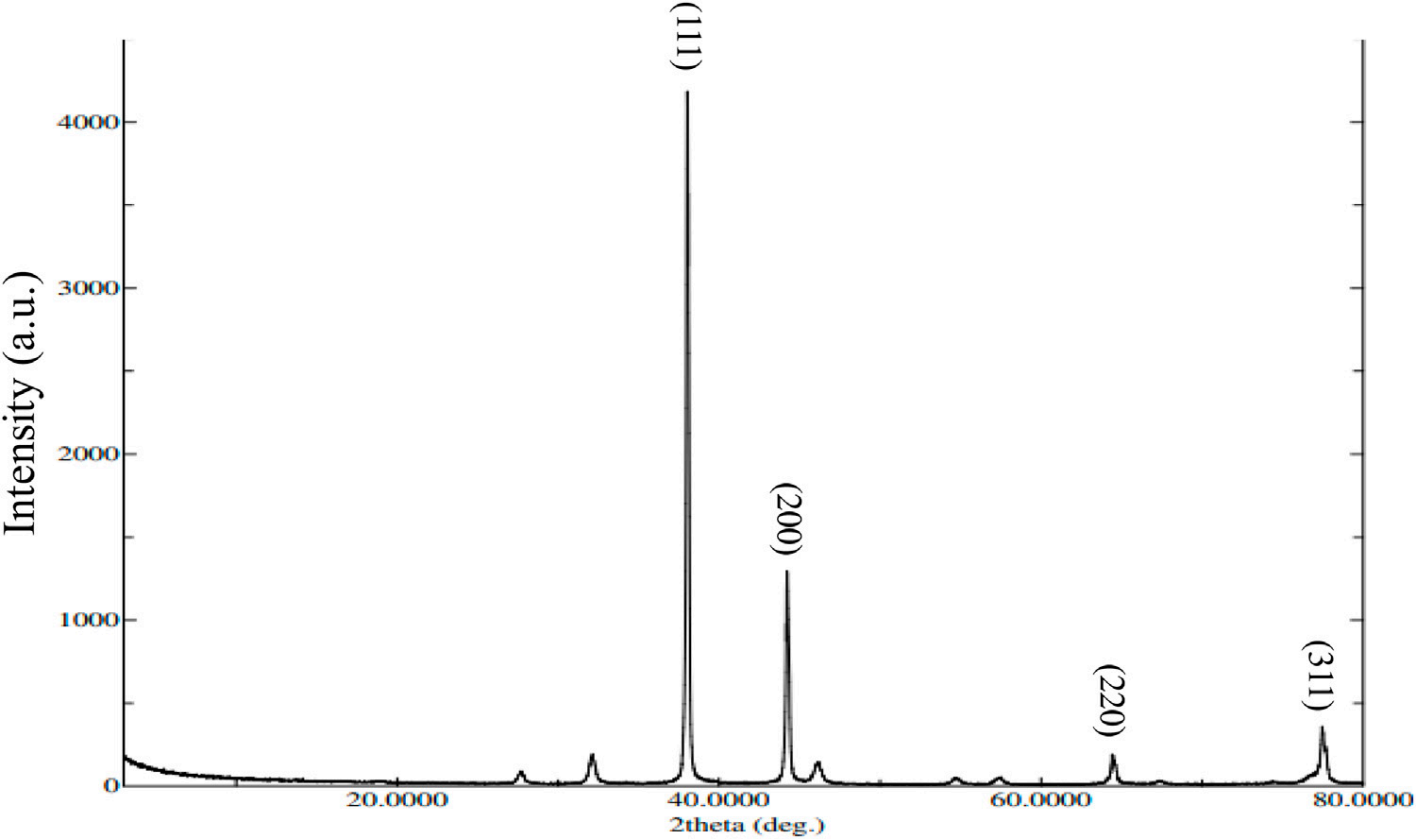
XRD pattern of green synthesized XT-AgNPs.

Figure 5 represents the XRD pattern of AgNPs, confirming their crystalline structure. The prominent diffraction peaks at 2θ values of 38.04°, 44.24°, 64.48°, and 77.48° are associated with the (111), (200), (220), and (311) planes of the face-centered cubic silver structure. In addition to these characteristic peaks, extra peaks were observed, which are attributed to the plant extract. These additional peaks could be because of organic compounds in extract that act by reducing silver ions and stabilizing the nanoparticles. Crystallite size (Dc) of the Ag nanoparticles was measured by Scherrer equation (Dc = Kλ/βcosθ), where β represent width of the diffraction peak at half intensity maximum, K represent shape factor (typically 0.9), and λ represent X-ray wavelength. The calculated crystallite size for XT-AgNPs was 27.90 nm, which is consistent with previously reported nanoparticle sizes (Atta et al., 2014).

#### SEM and TEM analysis

**FIGURE 6:**
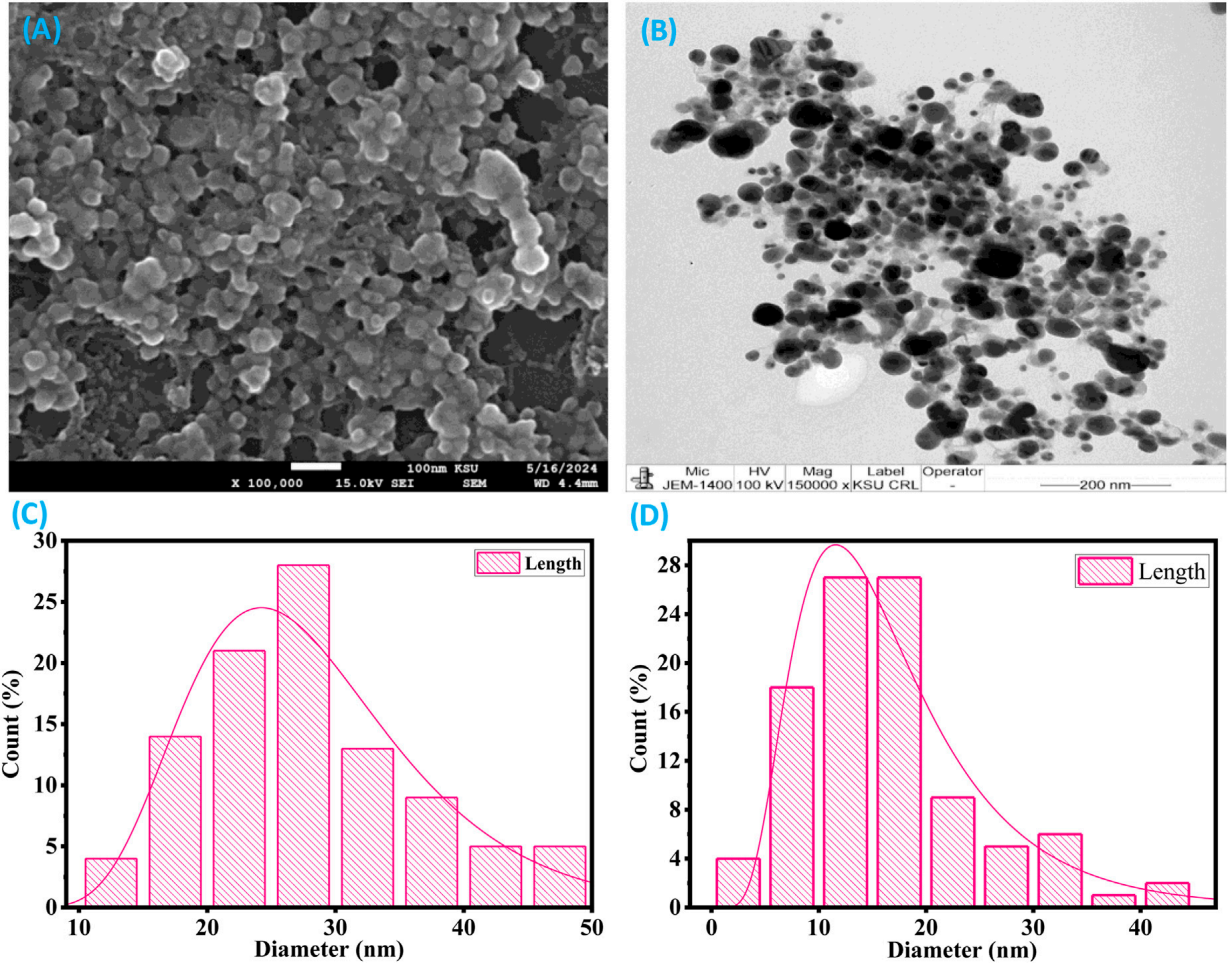
**(A)** SEM and **(B)** TEM images of green synthesized silver nanoparticles (XT-AgNPs). **(C)** and **(D)** are particle size distribution histogram plots analyzed using SEM and TEM images, respectively.

Scanning electron microscopy analysis of the XT-AgNPs revealed spherical particles with sizes below 100 nm, as shown in Figure 6A. Additionally, TEM showed detailed insights into the size and morphology of XT-AgNPs. These techniques have been frequently used by different workers (Francis et al., 2018) for characterization of nanoparticles. The TEM image shown in Figure 6B clearly illustrates that XT-AgNPs are spherical in shape. The image reveals agglomerates of small grains alongside some dispersed nanoparticles, corroborating the results obtained from SEM. The synthesized AgNPs were observed to have a size range of 10–50 nm. The size distribution of XT-AgNPs were also calculated based on the SEM and TEM images and the results are provided in Figures 6C,D.

### EDX

**FIGURE 7:**
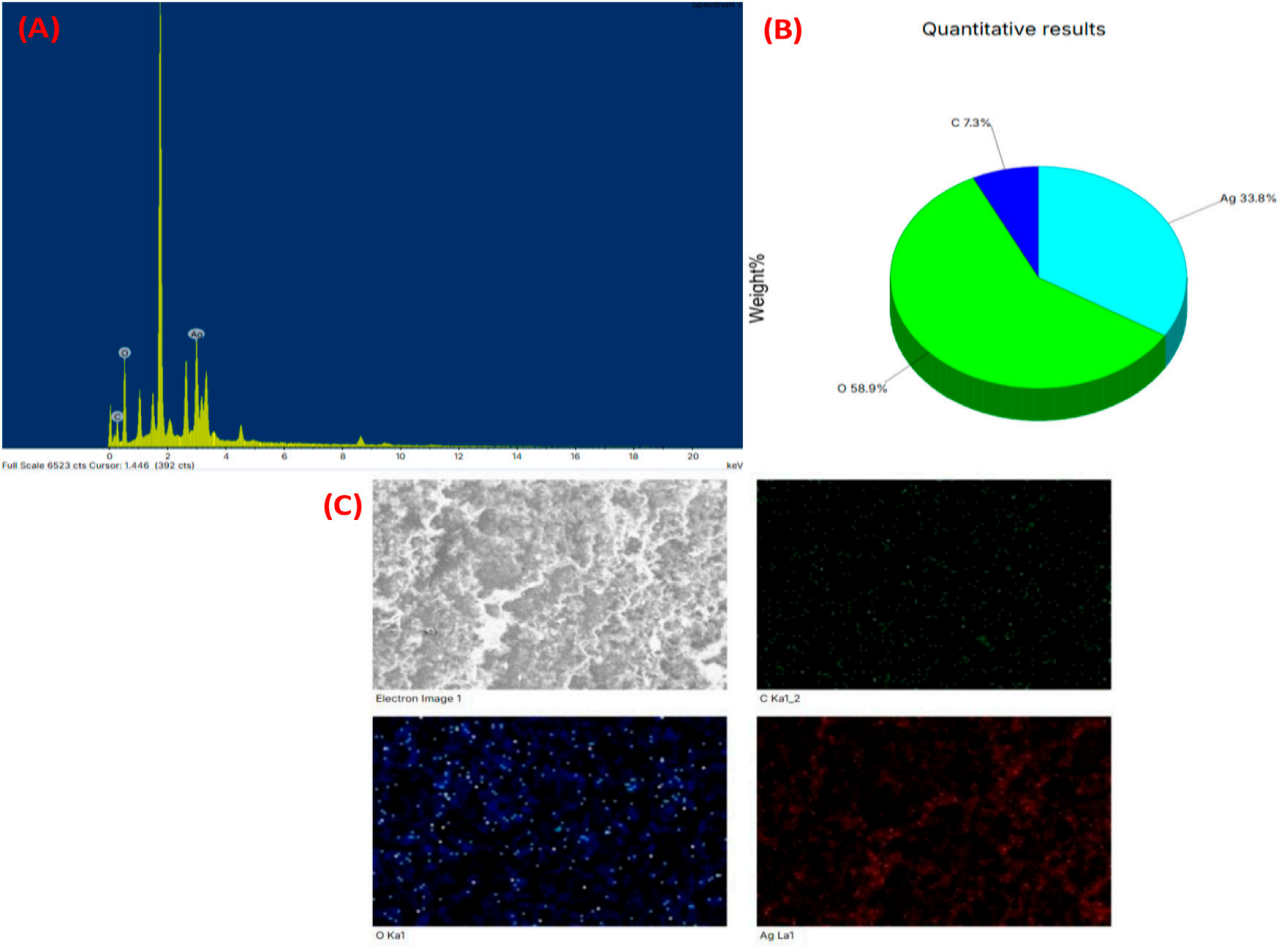
**(A)** EDX spectrum of silver nanoparticles synthesized (XT-AgNPs), **(B)** Quantitative analysis of elements present in it and **(C)** Elemental mapping of elements showing in XT-AgNPs.

Based on the EDX analysis, it was observed that there was a significant signal in silver region, indicating the occurrence of silver nanoparticles (as shown in Figure 7). Typically, AgNPs show an optical absorption peak around 3 keV, attributed to surface plasmon resonance, as reported previously by Magudapathy et al. (2001). The EDX analysis also demonstrated the presence of other elements which could be ascribed to the plant phytoconstituents that are capped on the surface of AgNPs.

### Zeta potential and DLS

**FIGURE 8:**
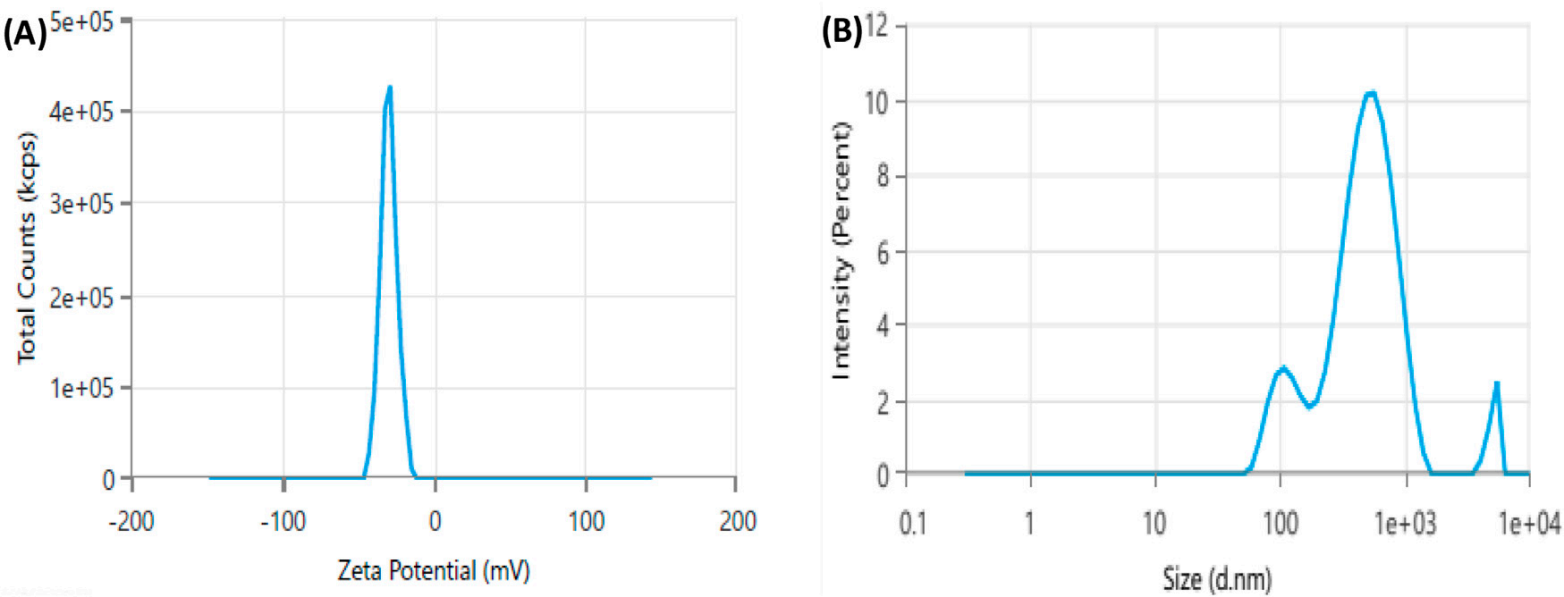
Dynamic light scattering (DLS) analysis of green synthesized silver nanoparticles (XT-AgNPs). **(A)** Zeta potential and **(B)** Size distribution.

Zeta potential (ζ) is a key parameter for assessing the stability of the synthesized nanoparticles in a solution, where nanoparticles with zeta potentials exceeding +30 mV or below −30 mV are highly stable in colloidal dispersions (Pompeu et al., 2018). Herein, XT-AgNPs zeta potential was measured at −29.65 mV (Figure 8A). This highly negative value indicates that the synthesized XT-AgNPs possess a high degree of stability. The distribution of XT-AgNPs particle size was analyzed with dynamic light scattering (DLS), a technique used to determine the hydrodynamic diameter of particles in a suspension, offering an approximate measurement of the average size of nanoparticles in the sample. The results showed that the XT-AgNPs had a size of 390 nm and a polydispersity index (PDI) of 0.46 (Figure 8B). Previous studies have stated that PDI indicates the uniformity of particles in a colloidal solution. This unitless value typically ranges from 0.05 to 0.7, in which a PDI value near 0.05 indicates that the particles are monodispersed, while values approaching 0.7 suggest a more heterogeneous particle distribution (Ghasemi et al., 2024). In present investigation, the PDI value of 0.46 is considered adequate. The difference in size observed between TEM (10–50 nm) and DLS (390 nm) is due to the fundamental differences in these techniques. While TEM reflects the actual core size of dried nanoparticles, DLS measures the hydrodynamic diameter in suspension, which encompasses the capping phytochemicals, solvation layer, and potential aggregation, resulting in a larger size value.

#### Anticancer activity of XT-AgNPs assessed by MTT and NRU assays

**FIGURE 9:**
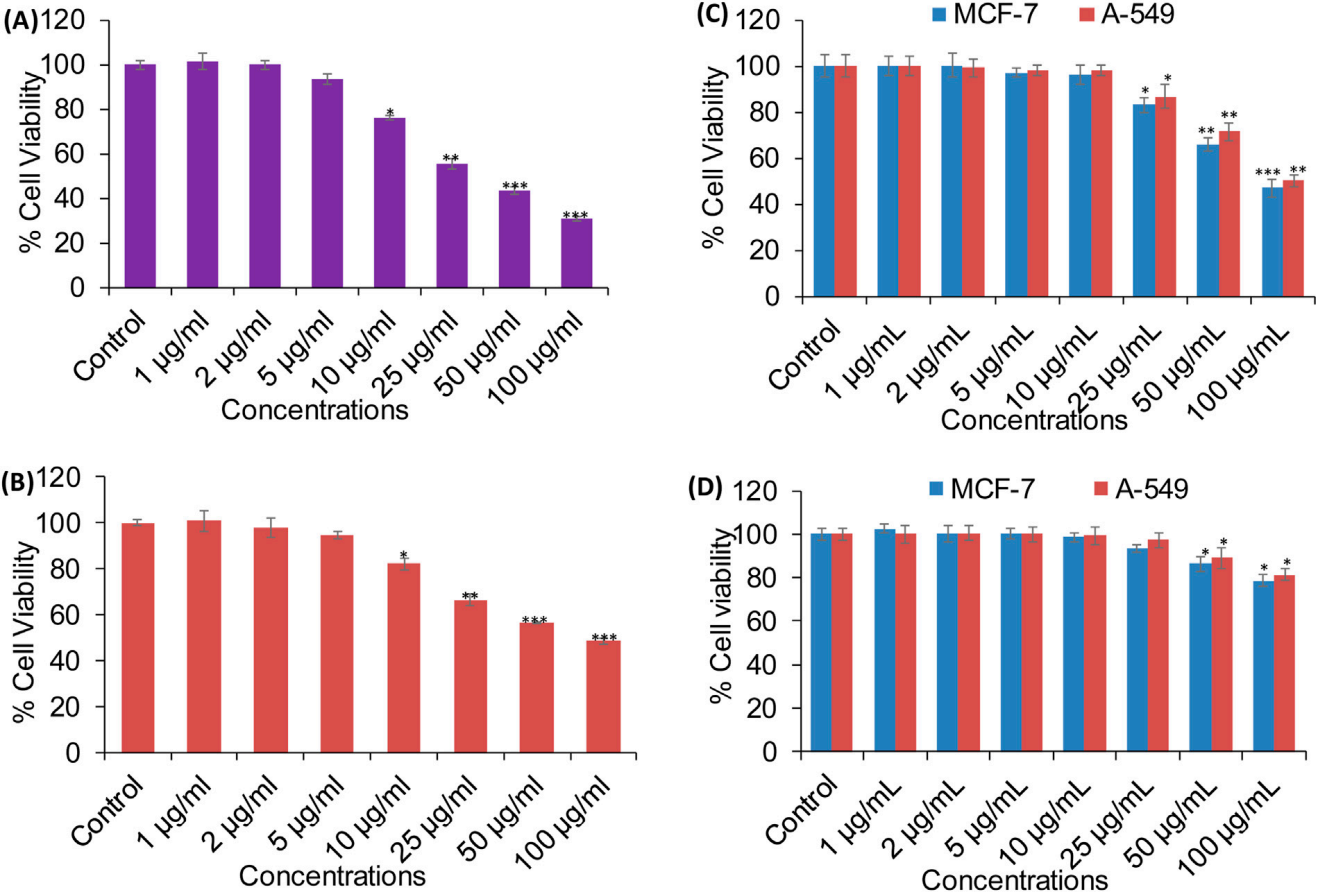
Cytotoxicity evaluation in **(A)** MCF-7 and **(B)** A-549 cells using the MTT assay after 24-hour exposure to varying concentrations of XT-AgNPs. **(C)** Cytotoxicity of commercial AgNPs and **(D)** XT extract in MCF-7 and A-549 cells. The data are presented as mean ± S.D. (n = 3). *p < 0.05, **p < 0.01, and ***p < 0.001 vs. control group.

A series of *in vitro* experiments were carried out to assess the biological efficiency of XT-AgNPs. The synthesized nanoparticles demonstrated cytotoxic effects on MCF-7 and A-549 cell lines in a dose-dependent way. Cell viability was significantly decreased even at a low concentration, i.e. 10 μg/mL of XT-AgNPs, indicating their high cytotoxicity against MCF-7 and A-549 cancer cells. The synthesized XT-AgNPs at 10, 25, 50, and 100 μg/mL reduced cell viability to 76%, 55%, 43%, and 30% for MCF-7 cells (Figure 9A) and 82%, 66%, 56%, and 48% for A-549 cells, respectively, compared to the untreated control (Figure 9B) by MTT assay.

Cytotoxicity was also evaluated using commercially available AgNPs (Sigma-Aldrich, USA). Our MTT data revealed that AgNPs induced a concentration-dependent reduction in cell viability, with an estimated IC_50_ values of 94 μg/mL for MCF-7 and 100 μg/mL for A-549 cells (Figure 9C). In comparison, *Xanthium strumarium* plant extract (XT extract) displayed relatively mild cytotoxicity, decreasing cell viability by only up to 22% in MCF-7 and 19% in A-549 cells at the highest tested concentration (100 μg/mL), with an IC_50_ value exceeding 100 μg/mL for both cell lines (Figure 9D). Collectively, these findings indicate that the phytochemicals present in the extract enhance the cytotoxic activity of biosynthesized XT-AgNPs compared with both commercial AgNPs and the extract alone, highlighting their promise as potential alternative anticancer agent.

**FIGURE 10:**
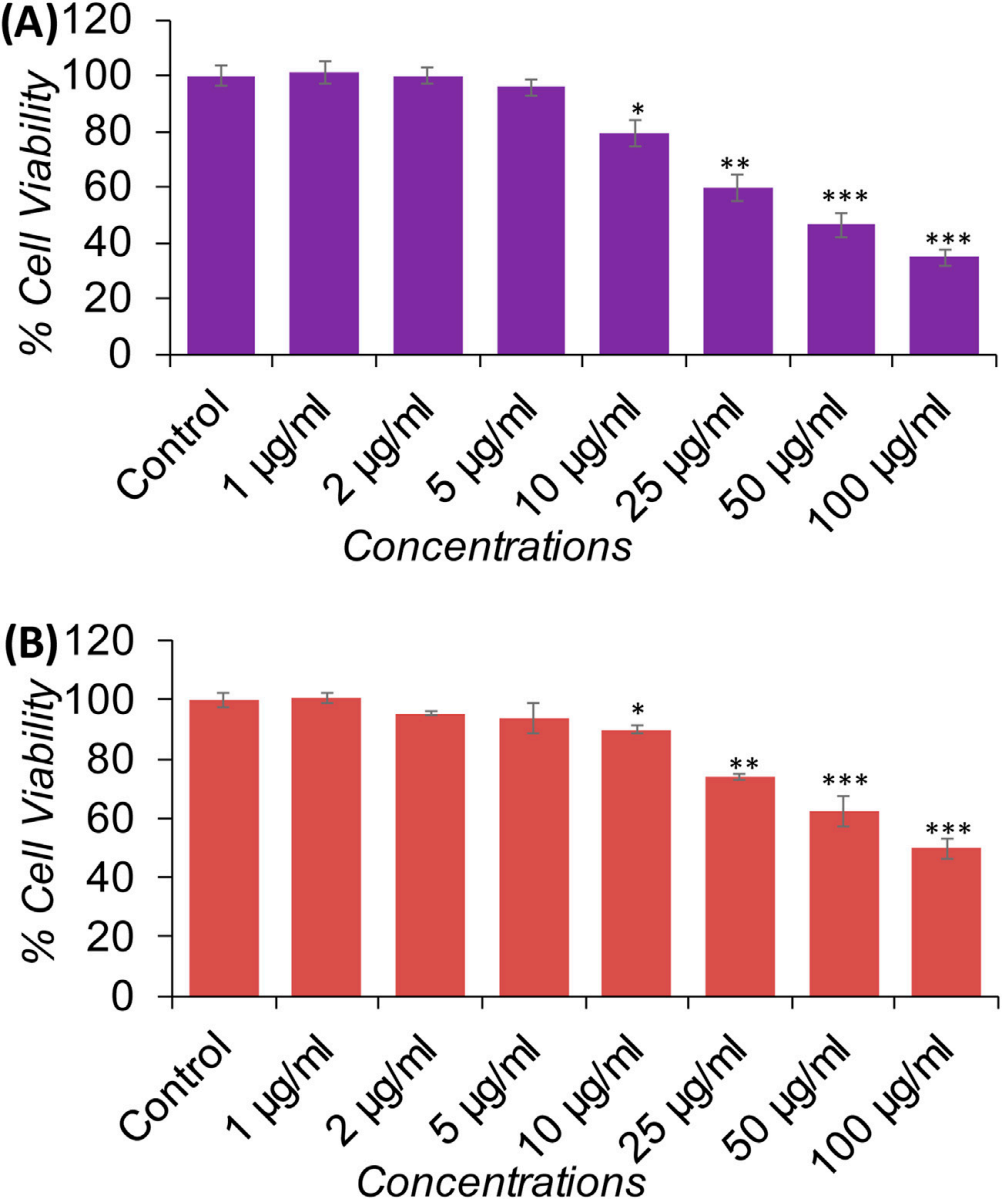
Cytotoxicity evaluation in **(A)** MCF-7 and **(B)** A-549 cells using the NRU assay after 24-hour exposure to different concentrations of XT-AgNPs. The data are presented as mean ± S.D. (n = 3) *p < 0.05, **p < 0.01, and ***p < 0.001 vs. control group.

In this study, NRU assay was also performed to measure the cytotoxicity caused by XT-AgNPs, which measure the lysosomal activity of the cells. The results showed that treatment with XT-AgNPs caused significant cytotoxicity on MCF-7 and A-549 cells, as evident by decreased cell viability. The cell viability in MCF-7 cells decreased to 79%, 59%, 46%, and 34% at 10, 25, 50, and 100 μg/mL of XT-AgNPs, respectively (Figure 10A). Similarly, in A-549 cells, the synthesized XT-AgNPs reduced cell viability to 89%, 73%, 62%, and 49% at 10, 25, 50, and 100 μg/mL respectively, relative to the untreated control (Figure 10B). As depicted in the figures, the IC_50_ values of XT-AgNPs against MCF-7 and A-549 cells were 44.3 μg/mL and 57.4 μg/mL, respectively for MTT assay. The IC_50_ values calculated for NRU assay were 46.6 μg/mL and 66.2 μg/mL against MCF-7 and A-549 cells, respectively.

These IC_50_ values clearly showed that the tested XT-AgNPs exhibited greater cytotoxicity towards MCF-7 cells than A-549, as evidenced by their effects on cellular metabolic activities. However, the NRU assay indicated slightly lower cytotoxicity compared to the MTT. In this study, the cytotoxicity of XT-AgNPs was assessed using two different assays. Moreover, the MTT assay results were correlated with those from the NRU assay. In the MTT assay, the accumulation of formazan serves as an indicator of mitochondrial activity in live cells, providing an indirect measure of cell viability (Van Meerloo et al., 2011). On the other hand, NRU assay evaluates lysosomal integrity by measuring the ability of viable cells to integrate the dye into organelles (Repetto et al., 2008). In contrast, our findings are aligned with those described by Al-Asiri et al. (2024), who found that the MTT assay demonstrated significant anticancer activity for green synthesized silver nanoparticles using *Euphorbia retusa* extract against human breast cancer cells MCF-7, with the IC_50_ values of 40 μg/mL. Similarly, Farshori et al. (2022) have reported that the green synthesized silver nanoparticle using *Phoenix Dactilyfera* showed a dose dependent cytotoxicity on human lung A-549 cells. They also stated that IC_50_ values for the NRU assay were greater than MTT assay as observed in current study. Our results were also supported by other investigators. Hublikar and colleagues have reported that green synthesized AgNPs from *Lantana camara* leaf extract exhibited anticancer efficacy against A-549 and MCF-7 cells with IC_50_ value of 49.52 μg/mL and 46.67 μg/mL, respectively (Hublikar et al., 2023).

#### Assessment of cell morphology

**FIGURE 11:**
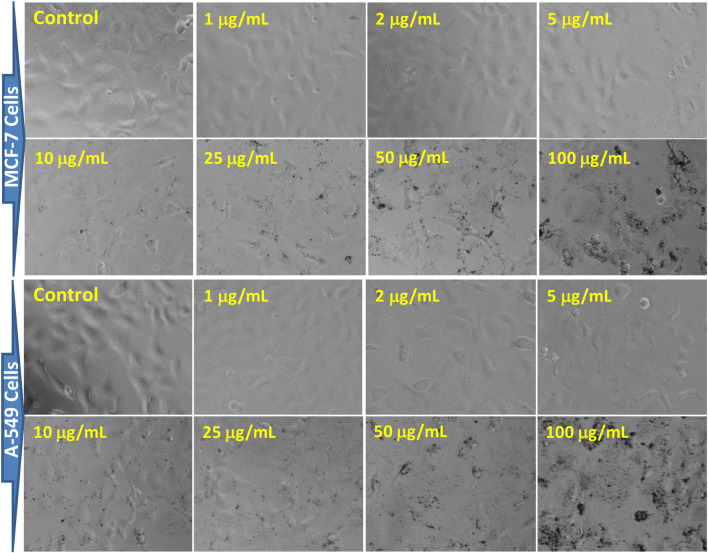
Morphological changes in MCF-7 and A-549 cells after 24 h of exposure to XT-AgNPs. The cell images were taken using a phase-contrast inverted microscope at 20x.

The morphology of both cell lines was significantly altered as the concentration of AgNPs increased (Figure 11). The cell images of both MCF-7 and A-549 showed that AgNPs at different concentrations impacted cell growth, with the effects being concentration-dependent. However, no significant morphological changes at the lower concentrations, i.e. 1–5 μg/mL were observed. The cytotoxicity of AgNPs, established by the MTT and NRU assays, was escorted by noticeable cell shrinkage, rounded bodies, and detachment, which was evident after the incubation period of 24 h with 10 μg/mL or higher concentrations of the green synthesized XT-AgNPs. The characteristic of cytoplasmic shrinkage is recognized as one of the initial events in cell death (Park et al., 2023). It plays a crucial role in eliminating dismissed and annoying cells in usual growth, as well as in the elimination of cancer cells induced by various agents (Pavlov et al., 2002), including green synthesized silver nanoparticles (Mittal et al., 2020). The cytomorphological changes observed in this study coincide with previous studies on the influence of green synthesized AgNPs on cytotoxicity of MCF-7 (Al-Sheddi et al., 2023) and A-549 cells (Farshori et al., 2022).

#### XT-AgNPs-induced intracellular ROS generation

**FIGURE 12:**
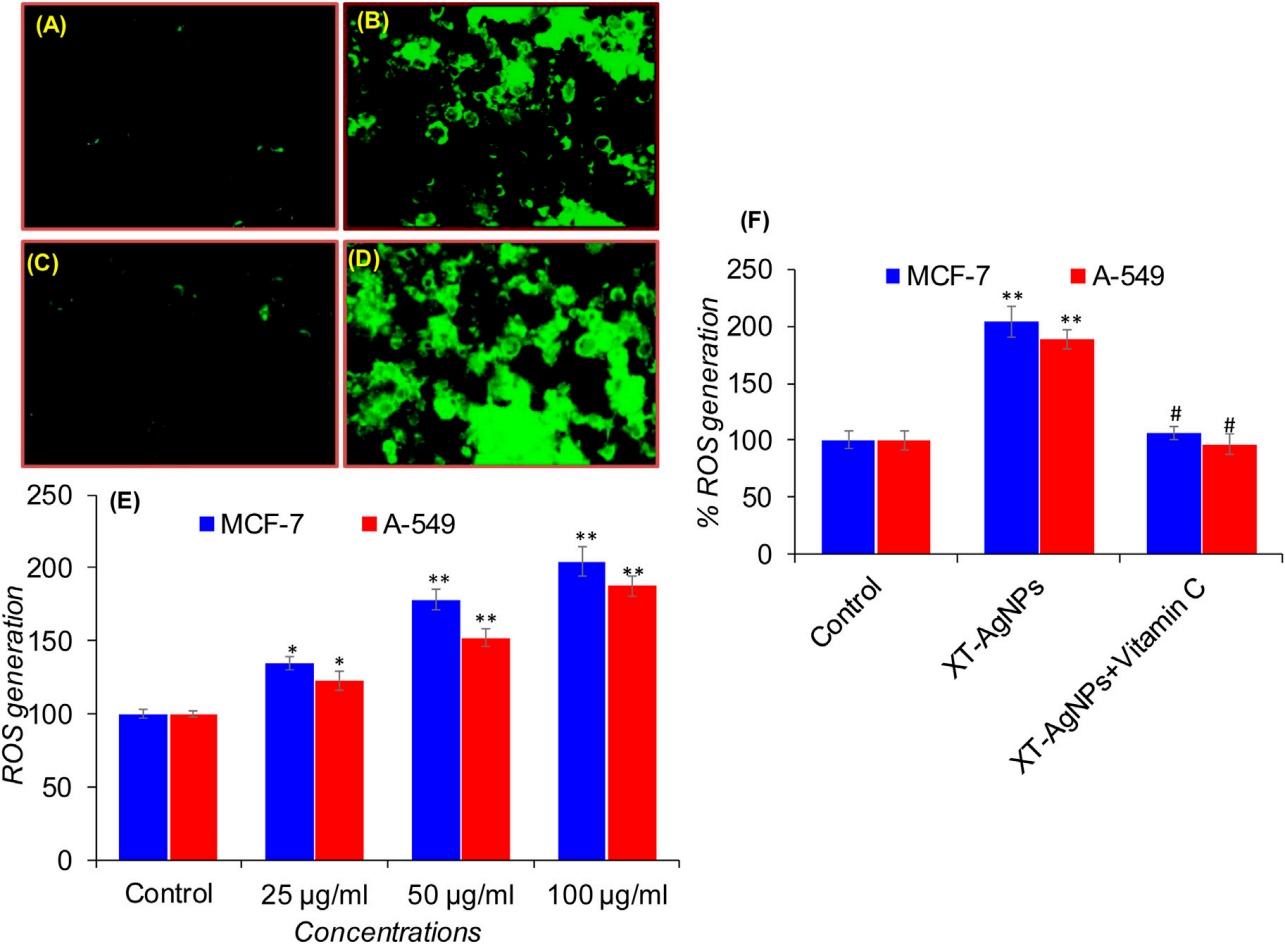
Green fluorescence indicating ROS production in MCF-7 and A-549 cells after XT-AgNPs exposure: **(A)** Control (MCF-7 cells), **(B)** 100 μg/mL of XT-AgNPs (MCF-7 cells), **(C)** Control (A-549 cells), **(D)** 100 μg/mL of XT-AgNPs (A-549 cells), and **(E)** Bar chart displaying the percentage change in ROS levels in MCF-7 and A-549 cells treated with 25–100 μg/mL of XT-AgNPs for 24 h. **(F)** Effects of co-treatment of Vitamin C (antioxidant; 1.5 mM) with or without XT-AgNPs (100 μg/mL) on ROS generation in MCF-7 and A-549 cells exposed for 24 h. The results are presented as mean ± S.D. *p < 0.05, **p < 0.01 vs. control and #p < 0.01 vs. XT-AgNPs treatment.

To explore mechanism(s) of cytotoxicity induced by XT-AgNPs in cancer cells, we further assessed intracellular ROS levels by determining DCF fluorescence in MCF-7 and A-549 cells exposed to XT-AgNPs at doses ranging from 25 to 100 μg/mL. Following treatment with XT-AgNPs, both cell lines showed a dose-dependent upsurge in ROS production. In MCF-7 cells, there was a noteworthy upsurge of 34%, 78%, and 104% in ROS generation compared to the control at 25, 50, and 100 μg/mL concentrations of XT-AgNPs (Figure 12). Likewise, A-549 cells exhibited 22%, 52%, and 87% increase in ROS production following XT-AgNPs exposure at same doses (Figure 12). These results highlight the significant ROS-producing potential of green synthesized AgNPs in both MCF-7 and A-549 cell lines.

Under physiological condition, maintaining the balance of redox system is crucial for normal function, and any disruption of this balance can result in oxidative stress (Schieber and Chandel, 2014). The interface amid AgNPs and mammalian cells may lead to oxidative stress by increasing ROS production beyond the cell’s antioxidant capacity (Kim and Ryu, 2013). The overproduction of ROS may also be a crucial factor in the cancer cell death induced by AgNPs treatment (Zhu et al., 2016). Various nanoparticles, including metal oxide particles, generate ROS as a key mechanism of cytotoxicity (Huang et al., 2010). Similarly, exposure to green synthesized AgNPs has been revealed to persuade cancer cell death through excessive ROS production in a number of cancer cells under *in vitro* conditions (Ullah et al., 2020; Al-Sheddi et al., 2023; Le et al., 2023). Our results have also shown that exposure to green synthesized XT-AgNPs trigger the production of ROS in MCF-7 and A-549 cells in a dose dependent way. These results uncover the molecular mechanism behind XT-AgNPs-induced ROS, which leads to apoptosis. It suggested that the green synthesized AgNPs-induced MCF-7 and A-549 cell death, as observed in this study, could be a result of excessive intracellular ROS generation, resulting in damage to cellular organelles such as mitochondria and lysosomes (He et al., 2024). We further explored the role of reactive oxygen species (ROS) in XT-AgNPs-induced cell death using Vitamin C, a potent ROS scavenger and antioxidant. To assess the contribution of ROS to XT-AgNPs-induced cytotoxicity, MCF-7 and A-549 cells were co-treated with XT-AgNPs (100 μg/mL) and Vitamin C (1.5 mM) for 24 h. The concentration of Vitamin C was selected based on earlier study (Ahamed et al., 2011). As illustrated in Figure 12F, treatment with XT-AgNPs markedly elevated intracellular ROS production in MCF-7 and A-549 cells. In contrast, co-treatment with Vitamin C efficiently suppressed ROS generation close to those of the control. These observations provide strong evidence that XT-AgNPs induce cell death in MCF-7 and A-549 cells primarily through ROS-mediated pathways.

#### *In silico* studies

##### Molecular docking

Molecular docking investigations were performed on phytocompounds and proteins involved in transcription regulation, DNA repair, and tumor suppression. In the current work, we chosen four (P^53^, BCl_2_, EGFR, and HER2) anticancer targets because they significantly influence apoptosis and begin signaling processes in carcinogenesis (Kalsoom et al., 2024). P^53^ is a pro-apoptotic protein that interacts with the anti-apoptotic protein BCL2. The interaction between BCL2 and P53 counteracts the inhibitory effect of BAX, releases cytochrome C, and ultimately triggers apoptosis (Salam et al., 2022).

**FIGURE 13:**
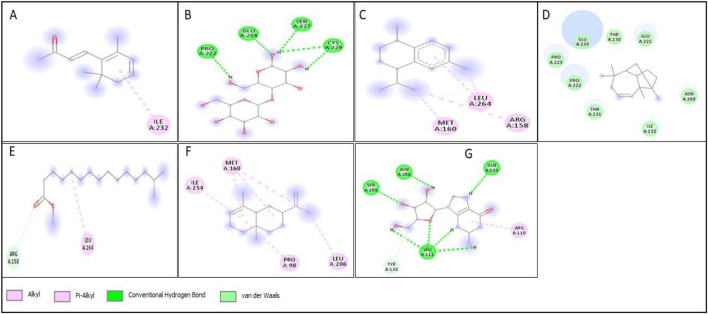
2D structure of interaction of compounds having maximum binding affinity at the active site of P53, **(A)** 4-(2,6,6-Trimethylcyclohexa-1,3-dienyl)but-3-en-2-one, Beta-D-Glucopyranose, **(B)** 4-O-Beta-D-galactopyranosyl, **(C)** 4-Isopropyl-1,6-dimethyl-1,2,3,4-tetrahydronaphthalene, **(D)** Longifolene-(V4), **(E)** Hexadecanoic acid, methyl ester, **(F)** Naphthalene, 1,2,3,4,4a,5,6,8a-oct ahydro-4a,8-dimethyl-2-(1-methylet henyl)-, [2R-(2.alpha.,4a.alpha.,8 a.beta.)]-, **(G)** Guanosine.

**FIGURE 14:**
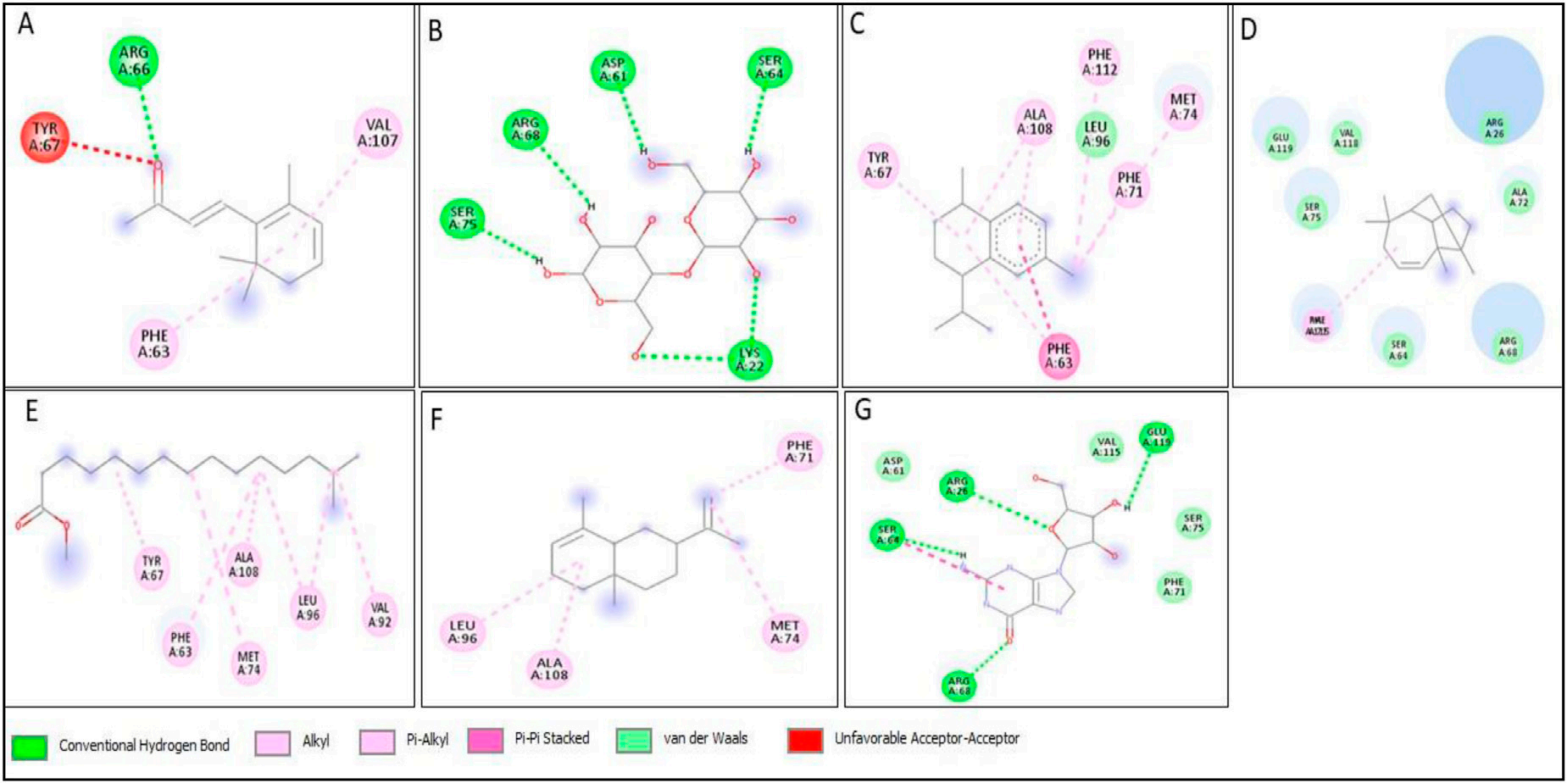
2D structure of interaction of compounds having maximum binding affinity at the active site of BCL2, **(A)** 4-(2,6,6-Trimethylcyclohexa-1,3-dienyl)but-3-en-2-one, Beta-D-Glucopyranose, **(B)** 4-O-Beta-D-galactopyranosyl, **(C)** 4-Isopropyl-1,6-dimethyl-1,2,3,4-tetrahydronaphthalene, **(D)** Longifolene-(V4), **(E)** Hexadecanoic acid, methyl ester, **(F)** Naphthalene, 1,2,3,4,4a,5,6,8a-oct ahydro-4a,8-dimethyl-2-(1-methylet henyl)-, [2R-(2.alpha.,4a.alpha.,8 a.beta.)]-, **(G)** Guanosine.

**FIGURE 15:**
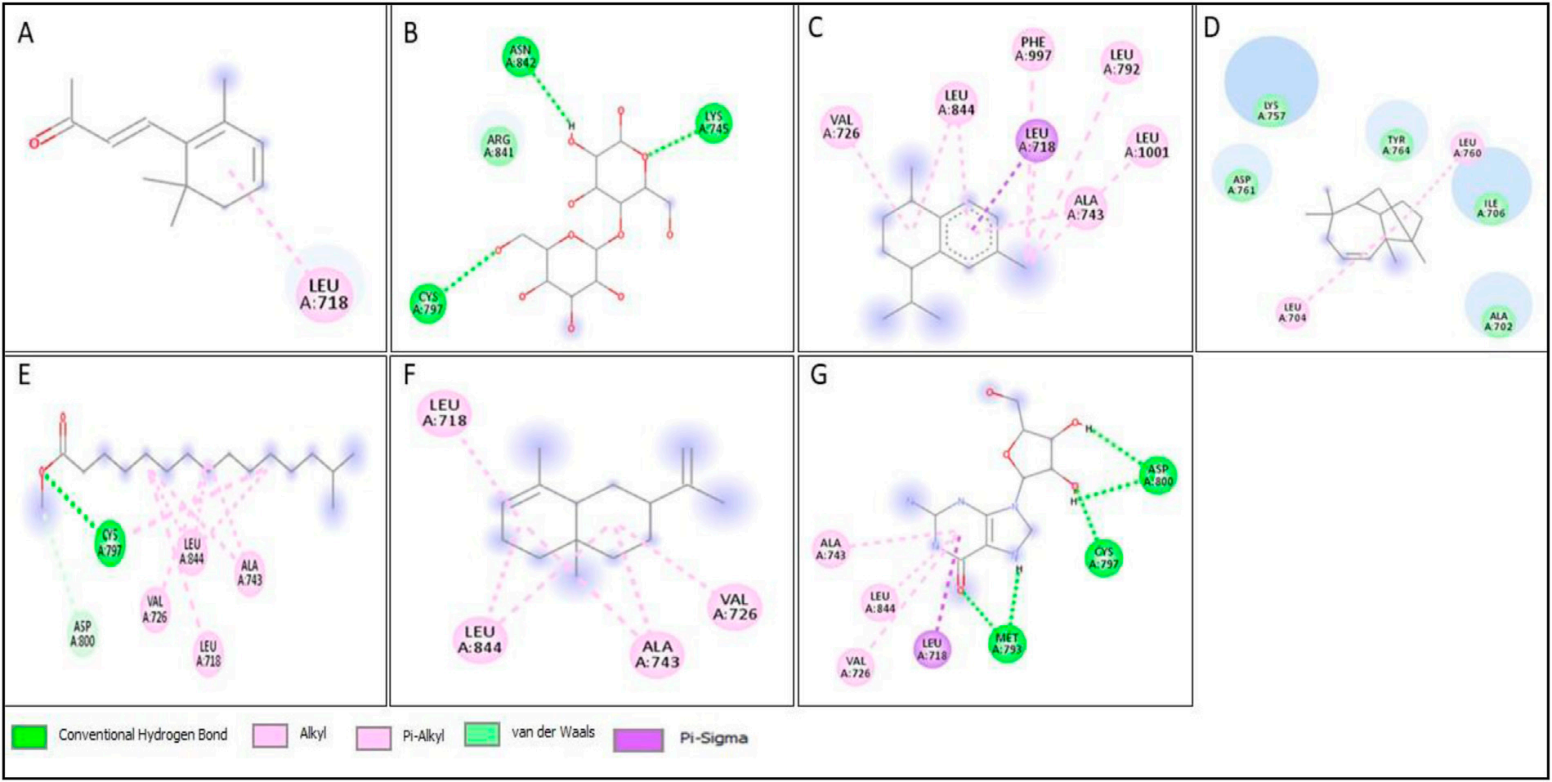
2D structure of interaction of compounds having maximum binding affinity at the active site of EGFR, **(A)** 4-(2,6,6-Trimethylcyclohexa-1,3-dienyl)but-3-en-2-one, Beta-D-Glucopyranose, **(B)** 4-O-Beta-D-galactopyranosyl, **(C)** 4-Isopropyl-1,6-dimethyl-1,2,3,4-tetrahydronaphthalene, **(D)** Longifolene-(V4), **(E)** Hexadecanoic acid, methyl ester, **(F)** Naphthalene, 1,2,3,4,4a,5,6,8a-oct ahydro-4a,8-dimethyl-2-(1-methylet henyl)-, [2R-(2.alpha.,4a.alpha.,8 a.beta.)]-, **(G)** Guanosine.

**FIGURE 16:**
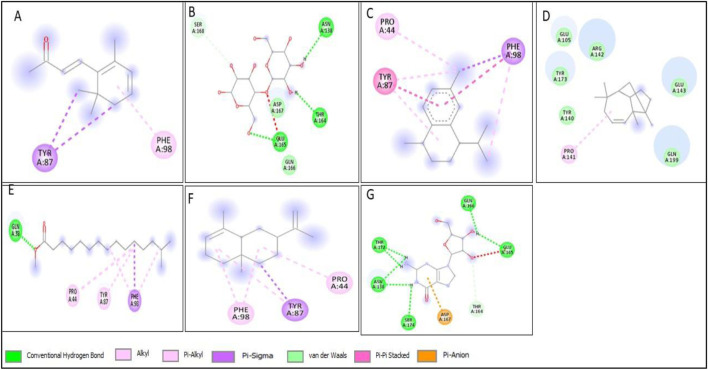
2D structure of interaction of compounds having maximum binding affinity at the active site of HER2, **(A)** 4-(2,6,6-Trimethylcyclohexa-1,3-dienyl)but-3-en-2-one, Beta-D-Glucopyranose, **(B)** 4-O-Beta-D-galactopyranosyl, **(C)** 4-Isopropyl-1,6-dimethyl-1,2,3,4-tetrahydronaphthalene, **(D)** Longifolene-(V4), **(E)** Hexadecanoic acid, methyl ester, **(F)** Naphthalene, 1,2,3,4,4a,5,6,8a-oct ahydro-4a,8-dimethyl-2-(1-methylet henyl)-, [2R-(2.alpha.,4a.alpha.,8 a.beta.)]-, **(G)** Guanosine.

All compounds tentatively identified by GC-MS were subjected to molecular docking against P53, BCL2, EGFR, and HER2. It was concluded that the binding affinities of seven compounds significantly exceeded those of the other compounds. However, the best compound was 4-Isopropyl-1,6-dimethyl-1,2,3,4-tetrahydronaphthalene, which demonstrated the highest activity among the tested compounds, showing a binding affinity of −7.5 with EGFR, which shows that this compound has the potential to interact with cancer-related proteins. While such interactions suggest possible mechanisms of action, it is important to note that these docking results represent hypothetical interactions and do not demonstrate that the compounds act in this way within a nanoparticle or cellular environment. We acknowledge that assuming phytoconstituents associated with the nanoparticle surface are available to interact with intracellular proteins in the same way as free molecules is a significant theoretical leap. The bioavailability, release kinetics, and stability of these bound phytoconstituents are unknown and likely differ from their unbound state. Therefore, the docking results should be considered only as supportive mechanistic hypotheses, useful for generating directions for further experimental validation, but not as direct evidence of anticancer activity. Nonetheless, the results provide valuable insight into potential protein interactions that warrant additional *in vitro* and *in vivo* testing. Result the seven phytoconstituents recognized from the GC-MS are described in Table 2. 2D structures of compounds having highest binding affinity against P53, BCL2, EGFR and HER2 are shown in Figures 13–16, respectively.

**TABLE 2:** Binding results of phytocompounds tentatively identified by GC-MS of extract of against P53, BCL2, EGFR and HER2.

SR #	Compound name	P53	Bcl2	EGFR	HER2
1	3-Hydroxy-N-methylphenethylamine	−5.1	−5.5	−5.9	−4.9
2	2,6-dimethoxy-phenol	−4.4	−4.7	−5.3	−4.7
3	4-(2,6,6-Trimethylcyclohexa-1,3-dienyl)but-3-en-2-one	−5.1	−6	−6.9	−6.0
4	Phenol	−4.1	−4.6	−4.8	−4.3
5	Hexanoic acid	−4.1	−4.4	−4.5	−4.4
6	Beta-D-Glucopyranose,4-O-Beta-D-galactopyranosyl	−5.8	−6.4	−6.5	−6.1
7	Stearic Acid	−4.4	−5.1	−6.2	−5.0
8	4-Isopropyl-1,6-dimethyl-1,2,3,4-tetrahydronaphthalene	−5.6	−7.3	−7.5	−5.9
9	Pilocarpine	−4.9	−5.8	−5.8	−4.6
10	Longifolene-(V4)	−5.4	−6.6	−6.3	−6.1
11	1-Octadecene	−3.4	−5.3	−5.8	−4.2
12	Neophytadiene	−4.5	−5.6	−6.6	−4.7
13	Tetradecanoic acid	−4.2	−5.2	−6	−4.5
14	Oxirane, hexadecyl-	−3.9	−5.2	−5.7	−4.2
15	2,4(1H,3H)-Pyrimidinedione, 5-methyl-	−4.6	−5	−5.5	−4.6
16	2-Cyclopenten-1-one, 3-methyl-2-(2,4-pentadienyl)-, (Z)-	−4.9	−5.9	−5.7	−5.3
17	Hexadecanoic acid, methyl ester	−5.7	−7	−7.4	−5.2
18	Pentadecanoic acid, 14-methyl ester	−4	−5.3	−5.2	−4.5
19	palmitic acid	−3.5	−5.3	−6.1	−4.4
20	p-Tyramine	−4.4	−5.3	−5.7	−4.5
21	Isopropyl Palmitate	−3.8	−5.1	−5.8	−3.8
22	cis-10-Heptadecenoic acid	−4.1	−5.5	−5.9	−4.6
23	9,12-Octadecadienoic acid (Z,Z)-,methyl ester	−4.2	−5.4	−6	−4.1
24	9,12,15-Octadecatrienoic acid, methyl ester, (Z,Z,Z)-	−4.2	−5.7	−7	−4.5
25	Phytol	−5	−5.4	−6.7	−4.6
26	Naphthalene, 1,2,3,4,4a,5,6,8a-oct ahydro-4a,8-dimethyl-2-(1-methylet henyl)-, [2R-(2.alpha.,4a.alpha.,8 a.beta.)]-	−5.4	−6.8	−7.1	−5.8
27	Guanosine	−5.9	−6.4	−6.5	−6.3

##### ADME

**FIGURE 17:**
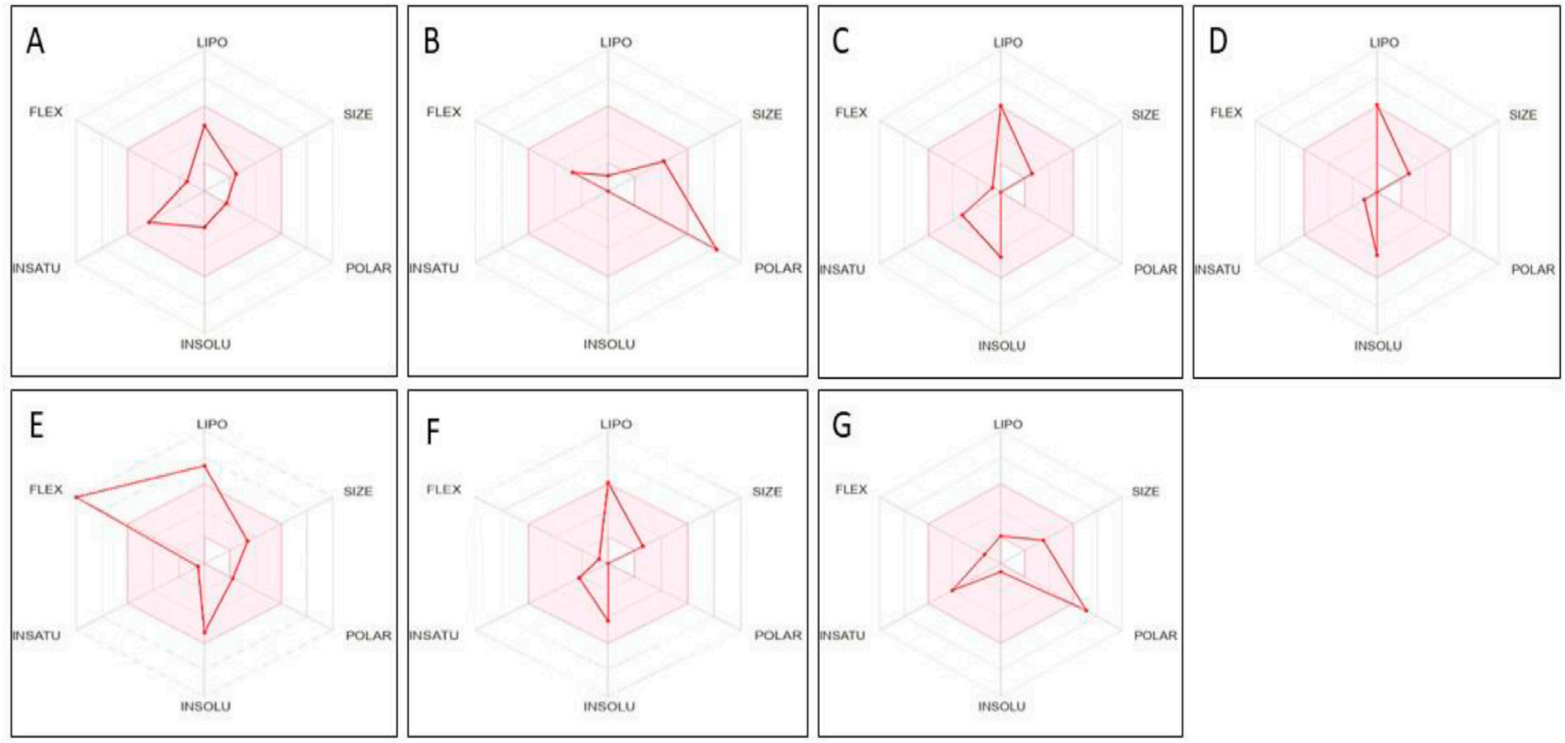
Bioavailability radar of compounds having maximum binding affinity at the active site of all tested proteins, **(A)** 4-(2,6,6-Trimethylcyclohexa-1,3-dienyl)but-3-en-2-one, Beta-D-Glucopyranose, **(B)** 4-O-Beta-D-galactopyranosyl, **(C)** 4-Isopropyl-1,6-dimethyl-1,2,3,4-tetrahydronaphthalene, **(D)** Longifolene-(V4), **(E)** Hexadecanoic acid, methyl ester, **(F)** Naphthalene, 1,2,3,4,4a,5,6,8a-oct ahydro-4a,8-dimethyl-2-(1-methylet henyl)-, [2R-(2.alpha.,4a.alpha.,8 a.beta.)]-, **(G)** Guanosine.

The seven phytoconstituents recognized from the GC-MS analysis of the extract were chosen based on their highest binding affinity (best docking score) and subsequently analyzed for ADME properties using the SWISS ADME online tool. This tool provides insights into pharmacokinetics, physical and chemical properties in addition to the drug-likeness characteristics of the top docked bioactive compounds. According to Lipinski’s Rule of Five, a compound that does not satisfy two or more of the rule’s criteria is considered unsuitable for oral administration. In this study, the ADME analysis of the selected compounds showed that four compounds violated one of Lipinski’s rules, while one compound, Beta-D-Glucopyranose, 4-O-Beta-D-galactopyranosyl, violated two rules, as shown in the table. Six out of the seven selected phytoconstituents are deemed suitable for oral administration, exhibiting characteristics of orally active drug-like compounds. Oral drug delivery systems offer significant advantages, including enhanced safety, improved patient compliance, pain avoidance, and benefits over other drug administration routes. Table 3 details the physicochemical properties of selected phytoconstituents, including lipophilicity, pharmacokinetic behavior, bond rotations, molecular weight, and the number of hydrogen bond donors and acceptors. Figure 17 illustrate the bioavailability radar of the selected compounds from the extract.

**TABLE 3:** Lipinski’s rule of five and solubility of best docked compounds.

SR NO	Best docked compounds	HBD	HBA	MWT	Lipophilicity	M.R	LR
1.	4-(2,6,6-Trimethylcyclohexa-1,3-dienyl)but-3-en-2-one	0	1	190.28	2.85	61.01	Yes; 0 violation
2.	Beta-D-Glucopyranose,4-O-Beta-D-galactopyranosyl	8	11	342.30	−4.37	68.12	No; 2 violations
3.	4-Isopropyl-1,6-dimethyl-1,2,3,4-tetrahydronaphthalene	0	0	202.34	5.45	68.07	Yes; 1 violation
4.	Longifolene-(V4)	0	0	204.35	5.65	66.62	Yes; 1 violation
5.	Hexadecanoic acid, methyl ester	0	2	270.45	4.44	85.12	Yes; 1 violation
6.	Naphthalene, 1,2,3,4,4a,5,6,8a-oct ahydro-4a,8-dimethyl-2-(1-methylet henyl)-, [2R-(2.alpha.,4a.alpha.,8 a.beta.)]-	0	0	204.35	4.63	68.78	Yes; 1 violation
7.	Guanosine	5	7	283.24	−2.76	65.50	Yes; 0 violation

HBD, hydrogen bond donor; MWT, molecular weight; Vn, violation, HBA, hydrogen bond acceptor; MR, molar refractivity.

##### Toxicity evaluation

The seven phytoconstituents recognized by GC-MS analysis were selected based on their highest binding affinity (optimal docking score) and subsequently assessed for toxicity using the PROTOX online tool. This tool provides information on predicted LD50, toxicity class, carcinogenicity, hepatotoxicity, mutagenicity, cytotoxicity and immunotoxicity of the selected phytoconstituents. All the compounds tested negative for hepatotoxicity, carcinogenicity, and cytotoxicity. However, 4-Isopropyl-1,6-dimethyl-1,2,3,4-tetrahydronaphthalene exhibited positive results for immunotoxicity and mutagenicity. Four of the phytoconstituents were classified under toxicity class 5. The toxicity evaluation results of the extract are presented in Table 4.

**TABLE 4:** Results of toxicity evaluation.

Sr no	Compound name	Predicted LD_50_ (mg/kg)	Predicted toxicity class	Hepatotoxicity	Carcinogenicity	Mutagenicity	Immunotoxicity	Cytotoxicity
1	4-(2,6,6-Trimethylcyclohexa-1,3-dienyl)but-3-en-2-one	5,000	5	_	_	_	-	_
2	Beta-D-Glucopyranose,4-O-Beta-D-galactopyranosyl	51	3	-	_	_	-	_
3	4-Isopropyl-1,6-dimethyl-1,2,3,4-tetrahydronaphthalene	6,700	6	_	_	+	-	_
4	Longifolene-(V4)	5,000	5	_	_	_	+	_
5	Hexadecanoic acid, methyl ester	5,000	5	_	_	_	-	_
6	Naphthalene, 1,2,3,4,4a,5,6,8a-oct ahydro-4a,8-dimethyl-2-(1-methylet henyl)-, [2R-(2.alpha.,4a.alpha.,8 a.beta.)]-	5,000	5	_	_	_	_	_
7	Guanosine	13	2	_	_	_	_	_

(+); Toxic, (−); Not Toxic.

## Conclusion

In conclusion, this study successfully demonstrated the effective biosynthesis of silver nanoparticles using *Xanthium strumarium* extract. XT-AgNPs showed a well-defined spherical shape, with average size of 27.90 nm. The synthesized XT-AgNPs demonstrated significant antiproliferative effects on human breast (MCF-7) and lung (A-549) cells in a concentration dependent way. Further the XT-AgNPs altered cancer cell morphology and enhanced the reactive oxygen species generation (ROS) upon 24 h of exposure. The findings from the current study confirm that XT-AgNPs induced cancer cell death was mediated through ROS generation, while the *in silico* results highlight hypotheses that must be validated through further *in vitro* and *in vivo* studies. Translating these findings into clinical applications therefore requires deeper investigation into the release, stability, and mechanistic pathways of XT-AgNPs in breast and lung cancer cells. Our findings also indicate that *Xanthium strumarium* mediated green synthesis of AgNPs is not only eco-friendly but also cost effective, as it relies on readily available plant resources and eliminates the need for hazardous chemicals. The phytochemical capping agents are likely to enhance durability and stability of the nanoparticles, making it suitable for scale-up.

## Data Availability

The original contributions presented in the study are included in the article/supplementary material, further inquiries can be directed to the corresponding authors.
